# Epigenetic predictors of all-cause mortality are associated with objective measures of neighborhood disadvantage in an urban population

**DOI:** 10.1186/s13148-020-00830-8

**Published:** 2020-03-11

**Authors:** Cavin K. Ward-Caviness, Shirley Pu, Chantel L. Martin, Sandro Galea, Monica Uddin, Derek E. Wildman, Karestan Koenen, Allison E. Aiello

**Affiliations:** 1 Center for Public Health and Environmental Assessment, US Environmental Protection Agency, 104 Mason Farm Rd, Chapel Hill, NC 27514 USA; 2 University of North Carolina–Chapel Hill, Chapel Hill, NC 27514 USA; 3 Carolina Population Center, Univeristy of North Carolina-Chapel Hill, Chapel Hill, NC 27514 USA; 4 Department of Epidemiology, Gillings School of Global Public Health, Univerity of North Carolina-Chapel Hill, Chapel Hill, NC 27514 USA; 5 School of Public Health, Boston University, Boston, MA 02118 USA; 6 Genomics Program, College of Public Health, University of South Florida, Tampa, FL 33612 USA; 7 Department of Epidemiology, Harvard T.H. Chan School of Public Health, Boston, MA 02115 USA

**Keywords:** DNA methylation, Social determinants of health, Neighborhood disadvantage, Mortality predictors, Urban populations

## Abstract

**Background:**

Neighborhood characteristics are robust predictors of overall health and mortality risk for residents. Though there has been some investigation of the role that molecular indicators may play in mediating neighborhood exposures, there has been little effort to incorporate newly developed epigenetic biomarkers into our understanding of neighborhood characteristics and health outcomes.

**Methods:**

Using 157 participants of the Detroit Neighborhood Health Study with detailed assessments of neighborhood characteristics and genome-wide DNA methylation profiling via the Illumina 450K methylation array, we assessed the relationship between objective neighborhood characteristics and a validated DNA methylation-based epigenetic mortality risk score (eMRS). Associations were adjusted for age, race, sex, ever smoking, ever alcohol usage, education, years spent in neighborhood, and employment. A secondary model additionally adjusted for personal neighborhood perception. We summarized 19 neighborhood quality indicators assessed for participants into 9 principal components which explained over 90% of the variance in the data and served as metrics of objective neighborhood quality exposures.

**Results:**

Of the nine principal components utilized for this study, one was strongly associated with the eMRS (*β* = 0.15; 95% confidence interval = 0.06–0.24; *P* = 0.002). This principal component (PC7) was most strongly driven by the presence of abandoned cars, poor streets, and non-art graffiti. Models including both PC7 and individual indicators of neighborhood perception indicated that only PC7 and not neighborhood perception impacted the eMRS. When stratified on neighborhood indicators of greenspace, we observed a potentially protective effect of large mature trees as this feature substantially attenuated the observed association.

**Conclusion:**

Objective measures of neighborhood disadvantage are significantly associated with an epigenetic predictor of mortality risk, presenting a potential novel avenue by which neighborhood-level exposures may impact health. Associations were independent of an individual’s perception of their neighborhood and attenuated by neighborhood greenspace features. More work should be done to determine molecular risk factors associated with neighborhoods, and potentially protective neighborhood features against adverse molecular effects.

## Background

The role of neighborhood characteristics in health cannot be overstated. Virtually, every characteristic of a neighborhood, here used broadly to define a relatively small area containing a residence, has been linked with the health and well-being of its residents including the chemical environment [1], the built environment [2, 3], the social and economic environment [4–6], and even individual perceptions of the neighborhood [7–9]. Though much of the existing research on the relationship between neighborhood characteristics and health has focused on overt health outcomes, like chronic disease and mortality, there is an increasing appreciation of the molecular alterations that may accompany residence in socioeconomically and built environment disadvantaged neighborhoods and that may explain the biological process through which exogenous factors like neighborhood characteristics “get under the skin.”

The stress pathway has been the most widely studied pathophysiologic pathway to explain how neighborhood characteristics impact health, as negative neighborhood characteristics are thought to chronically elevate stress levels. Allostatic load is a biomarker that incorporates multiple aspects of health, in particular stress-related health outcomes (e.g., blood pressure and cortisol), with higher allostatic load indicative of poorer health [10, 11]. Multiple studies have reported that allostatic load is increased for those living in stressful and socioeconomically disadvantaged neighborhoods [12, 13]. In addition to allostatic load, molecular markers of inflammation [14, 15] and accelerated aging [16, 17] are associated with exposure to adverse chemical and socioeconomic environments. In the reverse of these studies, neighborhood factors associated with positive health outcomes, e.g., greenspace, have been associated with improved levels of molecular biomarkers for allostatic load and inflammation [18], demonstrating that both positive and negative neighborhood characteristics might impact molecular indicators of health.

One of the most promising molecular markers to study health impacts is DNA methylation. DNA is primarily methylated at cytosine of cytosine-phosphate-guanine (CpG) dinucleotides. DNA methylation is a central process by which the expression of genes is regulated in both a cell type-specific and temporal manner [19]. DNA methylation altered by exposure to both the chemical and non-chemical (i.e., socioeconomic) environment, and alterations in DNA methylation are associated (in some cases with causal evidence) with a variety of health outcomes including metabolic outcomes [20, 21], cardiovascular disease [22–24], cancer [25, 26], and mortality [27, 28].

Recent advances in DNA array technology and data availability have allowed researchers to develop DNA methylation-based biomarkers for outcomes such as accelerated aging [29], chronic disease risk [30], and mortality risk [27, 31]. For aging, there has been substantial work showing that DNA methylation age is accelerated by adverse environmental exposures [17]. For other epigenetic biomarkers, there has been little research to evaluate their response to the external environment, in particular neighborhood characteristics. Here, we evaluate the degree to which objective measures of neighborhood quality/disadvantage, such as the condition of the street and buildings and lack of greenspace, are associated with a validated epigenetic mortality risk score (eMRS) [27, 31]. We further do this in a predominantly African-American cohort, an understudied racial group in the field of epigenetics.

## Results

For the 157 participants in this analysis, the mean age was 53.3 years (standard deviation = 13.8 years), 61.8% (97) were female, and 87.9% were African-American. At initial enrollment, 20.4% (32) of the participants in this analysis reported never smoking, and 47% (74) had at least some college education (Table 1). At the baseline assessment, 126 participants (80.3%) reported that they liked their neighborhood either “somewhat” or “a great deal” as a place to live.

**Table 1:** Description of Detroit Neighborhood Health Study participants used in this analysis

Cohort description	Mean	SD
Age (years)	53.3	13.8
Years lived in neighborhood (years)	18.6	16.9
B cell (%)	8.12	3.93
Monocytes (%)	8.64	2.97
Granulocytes (%)	53.5	11.7
CD4-T cells (%)	16.9	7.42
CD8-T cells (%)	10.7	6.13
Natural killer cells (%)	7.85	5.85
	*N*	%
Sex (female)	97	61.8
Never smokers	43	27.4
Never drinkers	32	20.4
Education (kindergarten–eighth grade)	2	1.27
Education (some high school)	26	16.6
Education (high school equivalent)	14	8.92
Education (high school graduate)	41	26.1
Education (some college)	46	29.3
Education (college graduate)	20	12.7
Education (post-graduate degree)	8	5.1
Employed	46	29.3
Close knit neighborhood (strongly or somewhat agree)	87	55.4
Like the Neighborhood (somewhat–a great deal)	126	80.3
White	17	10.8
Black or African-American	138	87.9
Other	2	1.27

Percentages of cell types were estimated from the DNA methylation data as described in the “Methods” section

**Fig. 1:**
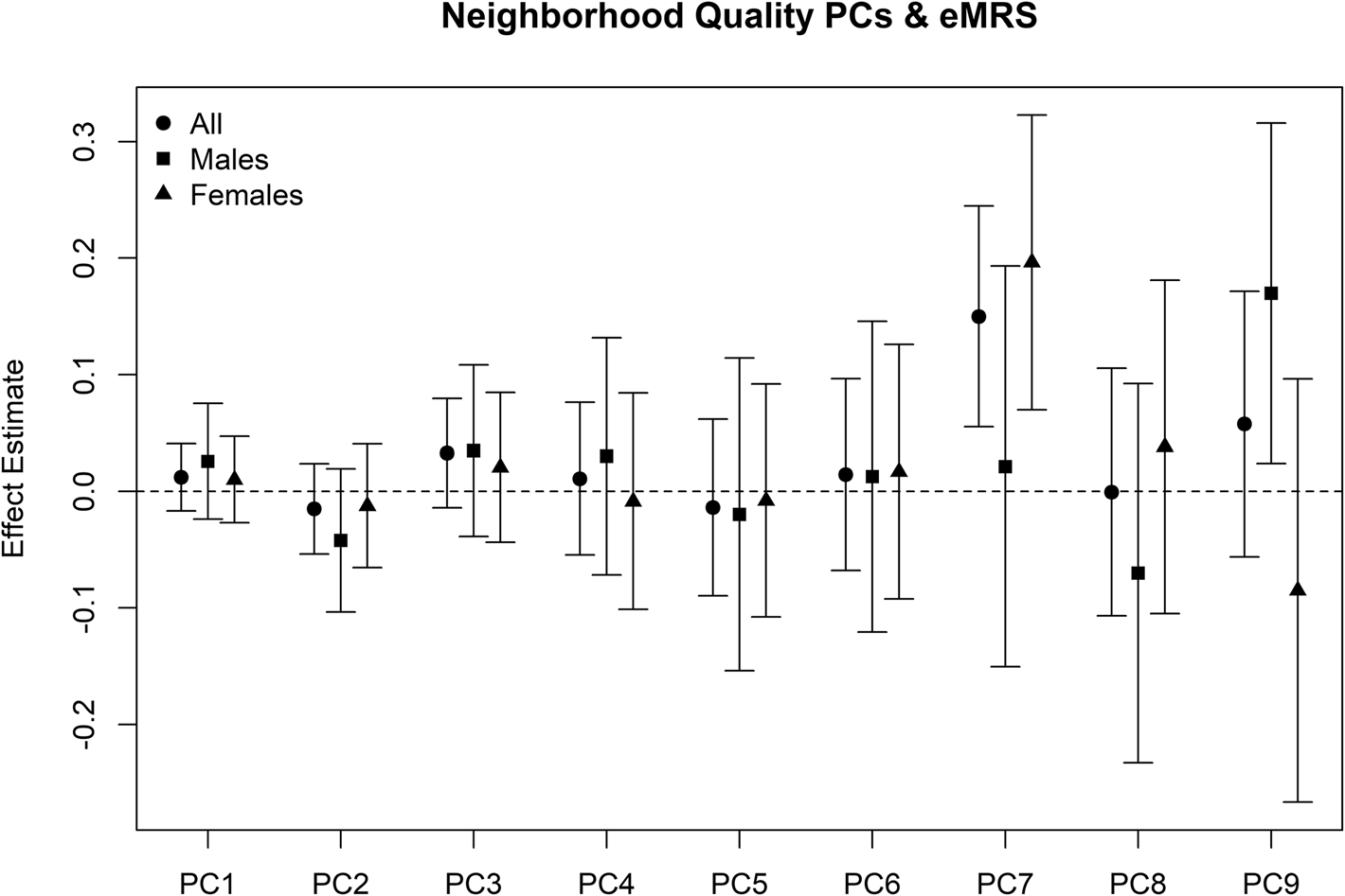
Association between principal components for neighborhood quality and eMRS. Each of the principal components (PCs) for neighborhood quality was associated with the epigenetic mortality risk score (eMRS [27]) in a model adjusted for age sex, race (White, African-American, and Other), ever smoking, ever alcohol usage, years spent residing in neighborhood, education (binary indicator for some college or more), and employment (binary indicator for employed vs unemployed). Squares and triangles represent models stratified on sex (males and females, respectively), and in such models, the term for sex was removed. Principal components are numbered in order of their ranking of the percent variance explained, and only the top nine were examined as they explained > 90% of the variance as detailed in the “Methods” section

Of the nine PCs, only one (PC7) was associated with the eMRS (Fig. 1) in a model adjusted for age, sex, race, smoking status, alcohol usage, years residing in the neighborhood, education, and employment after correcting for the nine associations performed in the primary analysis. The association remained in a model that further adjusted for individual perception of the neighborhood, and an individual’s perception of their neighborhood was not independently associated with the eMRS in the model containing PC7 (Table 2). The association appeared to be primarily driven by female participants (Fig. 1). Blood samples for the participants in this study came from two survey waves. Of the 157 participants, 111 (71%) came from wave 2. When stratifying on wave 2 participants, we observed minimal change in the association with PC7 (effect estimate = 0.16; 95% CI = 0.05–0.26; *P* = 0.004). The top positive loadings for PC7 were factors associated with the presence of abandoned cars, people present on the street, and non-art graffiti. In addition, the presence of alcohol advertising had a negative loading, as well as the street being in poor condition (Supplemental Table 1).

**Table 2:** Assessment of both objective and subjective neighborhood measures in association with epigenetic mortality risk score

Variable	Effect estimate	95% CI	*P* value
PC7	0.15	0.06, 0.25	0.002
Close knit neighborhood	0.08	− 0.07, 0.23	0.30
Good place to live	0.01	− 0.18, 0.20	0.93

In a model which assessed both objective measures of neighborhood quality (neighborhood principal component 7 [PC7]) as well as subjective measures of neighborhood quality (whether the neighborhood was close knit and whether the neighborhood was a good place to live according to study participants), only the objective measure was associated with the epigenetic mortality risk score [27]. Effect estimates are given in per unit increases in the epigenetic mortality risk score per a one unit increase in the neighborhood quality principal component

In a sensitivity analysis, none of the individual neighborhood characteristics were associated with the eMRS in isolation after correcting for the 19 associations performed. Only one, HQ11 indicating if the streets in the neighborhood are in poor condition, had even a nominal (*P* < 0.05) association (Supplemental Table 3). This suggests that associations between neighborhood features and the eMRS are strongest when multiple features are combined.

PC7 was associated with 4 of the 10 CpGs composing the eMRS at *P* < 0.005 (which adjusts for the 10 associations performed in this sensitivity analysis). For all but one of the PC7-CpG associations with *P* < 0.005, the direction of association was consistent with the eMRS-PC7 association after accounting for the direction of association for the CpG with the eMRS observed by Zhang et al. [27] (Table 3). This indicates that PC7 may be associated with multiple epigenetic loci that are predictive of mortality, an association which is even stronger (by effect size) when aggregated into an eMRS.

**Table 3:** Association between PC7 and components of the eMRS

	Beta	*P* value	95% CI	Chr	Gene
cg08362785	0.01	4.0 × 10^−4^	0.006, 0.02	22	*MKL1*
cg01612140	− 0.03	4.5 × 10^−4^	− 0.04, − 0.01	6	
cg23665802	− 0.02	0.001	− 0.03, − 0.008	13	*MIR19A*
cg24704287	− 0.02	0.002	− 0.03, − 0.007	19	
cg25983901	− 0.01	0.02	− 0.02, − 0.003	7	
cg10321156	− 0.01	0.02	− 0.03, − 0.002	11	
cg19572487	0.01	0.23	− 0.005, 0.02	17	*RARA*
cg06126421	− 0.01	0.26	− 0.03, 0.007	6	
cg14975410	− 0.002	0.70	− 0.02, 0.01	3	
cg05575921	0.001	0.88	− 0.02, 0.02	5	*AHRR*

Multiple DNA methylation loci (CpG) which composed the epigenetic mortality risk score (eMRS) even after a multiple test correction for the 10 tests performed (*P* < 0.005). Chromosome and associated gene were taken from the Illumina 450K manifest file. Gene annotation is by proximity. *CI* confidence interval; *Chr* chromosome

When stratified by sex, the associations appeared to be driven by women (Fig. 1). Although PC9 also appeared to have a sex-specific association, we did not consider this in the sex-specific analysis as there was no PC9-eMRS association in the primary analysis, and even the sex-specific association would not have been significant after adjusting for the number of tests performed.

**Fig. 2:**
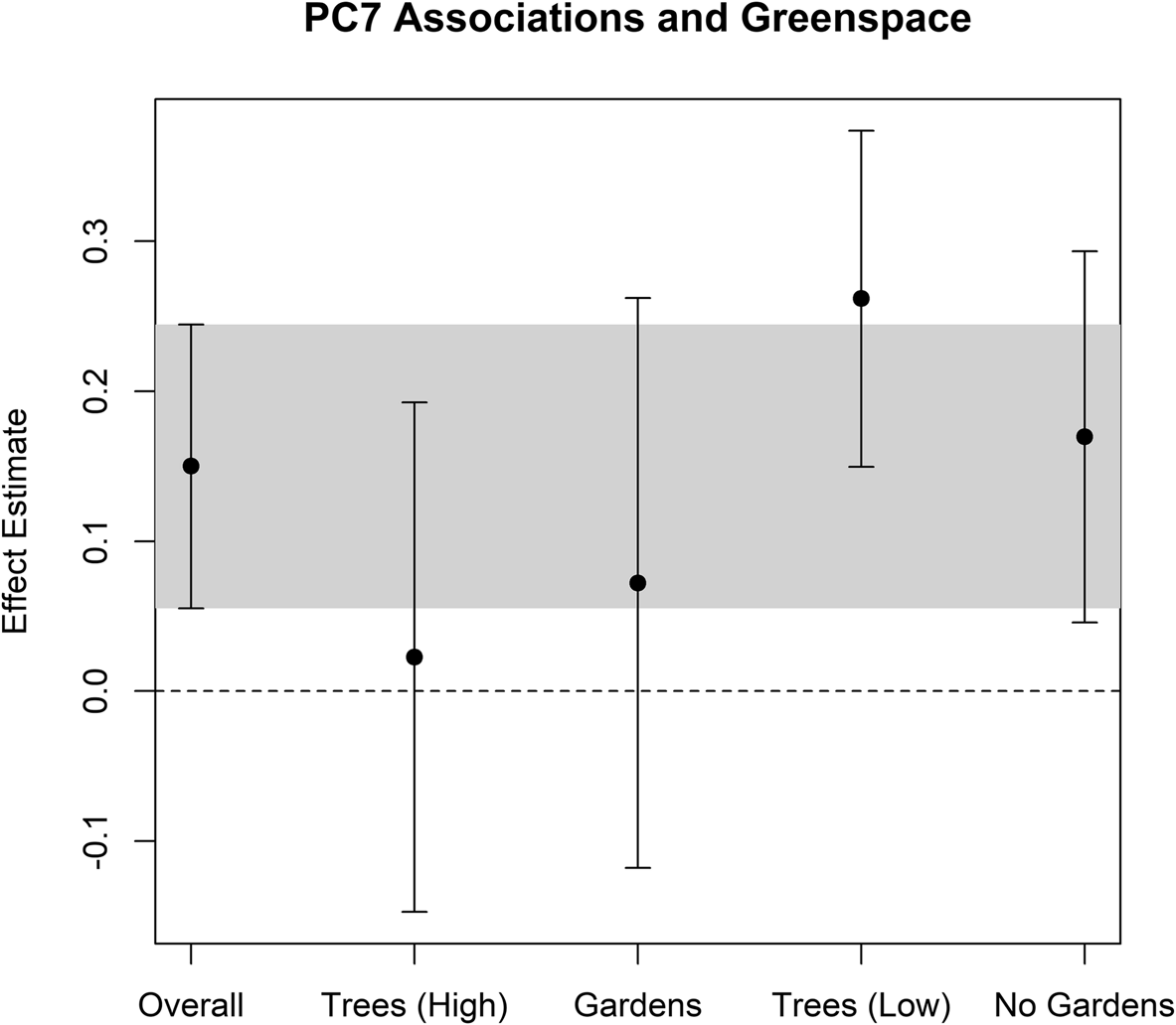
Association between PC7 and eMRS stratified on indicators of neighborhood greenspace. To examine if associations were potentially modified by exposure to greenspace, we stratified associations between neighborhood principal component 7 (PC7) and the epigenetic mortality risk score (eMRS [27]). Associations appeared to be substantially stronger in neighborhoods with greater than the median presence of large mature trees (Trees (High)) and with community gardens (Gardens) as compared to neighborhoods with below median presence of large mature trees (Trees (Low)) and no observed community gardens (No Gardens). The median presence of large mature trees was 84.2%. Gray bar indicates region as defined by the 95% confidence interval for the overall association

The presence of greenspace has been implicated as a potentially protective environmental factor for health outcomes [2, 32]. The presence of large, mature, trees and the presence of community gardens were two assessed neighborhood characteristics that could speak to the presence of greenspace, and both were uncorrelated with PC7 (Pearson’s *r*^2^ = 0.004 and 0.001, respectively). To evaluate the impact of greenspace, we examined if associations between PC7 and the eMRS were attenuated in individuals residing in neighborhoods where the percentage of street segments with large mature trees was above the median (> 84.2%) or in neighborhoods with at least one community garden observed. Indeed, individuals residing in neighborhoods with above median levels of large mature trees saw an 85% attenuation of the PC7-eMRS association as compared to the entire cohort (Fig. 2). Conversely, the PC7-eMRS association was elevated for individuals with below median levels of large mature trees as compared to the overall cohort. For community gardens, we observed a smaller attenuation towards the null (52%) when comparing communities with no community gardens to those with one or more. The 95% confidence intervals of the stratified associations for community gardens largely overlapped indicating a weaker attenuation than seen with large mature trees which should be regarded cautiously given the small sample sizes (Fig. 2). The attenuation of associations in neighborhoods with greenspace extended even to the individual CpGs which compose the eMRS, as associations were substantially attenuated for individuals living in neighborhoods with above median levels of large mature trees or community gardens as compared to individuals living in neighborhoods with below median levels of large mature trees or no community gardens (Supplemental Table 4). Inflammation may be one mechanism by which the external environment is associated with epigenetic mortality risk. Blood immune cell counts can be used to examine the impact of inflammation on associations, which would not be considered confounders here (as they might in an epigenome-wide association study) as inflammation is a mechanism by which the exposure may act on the outcome. In Supplemental Table 5, we show the Pearson correlation (*r*^2^) between each principal component and six cell counts (estimated from DNA methylation data using the Houseman method). For PC7, correlations were fairly weak and ranged from 5.9 × 10^−5^ to 0.07. Adjusting for cell counts reduced the overall association as well as for neighborhoods with community gardens. Associations were still present for neighborhoods without large mature trees (Supplemental Table 6), indicating that while inflammatory status likely plays a role in associations, at least for neighborhoods without large mature trees, there persists a component likely independent of inflammation as reflected in changes in blood immune cell counts.

## Discussion

Using data from residents of Detroit, we find that a recently developed epigenetic biomarker of mortality [27] is associated with summary measures of neighborhood characteristics constructed from objective measures of neighborhood characteristics which are indicative of neighborhood advantage/disadvantage based primarily on the quality of the built environment (Supplemental Table 2). The continuous epigenetic mortality score developed by Zhang et al. [27] is a strong predictor of mortality, and in an independent validation study, a one unit increase was associated with a 2.64-fold increase (95% confidence interval, 1.98–3.52) in all-cause mortality risk [31]. We found that PC7, a principal component driven by neighborhood characteristics such as the number of abandoned cars observed, people being present outside on the street, and non-art graffiti (Supplemental Table 1, Supplemental Table 2), was associated with higher epigenetic risk of mortality (Fig. 1).

Greenspaces have been promoted as a protective environmental measure against adverse health outcomes [2, 32, 33], and may even be able to attenuate the effect of other adverse chemical or social environmental exposures. In a study of more than 40,000,000 adults, the association between all-cause mortality and income deprivation was lowest in the most green areas [34]. The presence of large, mature trees substantially attenuated the association between PC7 and the eMRS, indicating a potentially counteractive effect of greenspace indicators against the adverse community factors represented by PC7. We saw less attenuation with stratification on the presence of community gardens, and the differences in the observed associations may have been driven by chance. However, these stratified analyses should be considered preliminary and interpreted with a note of caution given the small sample sizes and overlapping confidence intervals for the analysis of community gardens in particular. We also cannot discount that the presence of community gardens may not only be reflective of increased greenness in a neighborhood, but might also reflect altered dietary conditions through access to fresh foods.

While there have been multiple studies linking area-level neighborhood features (or correlates of these features) with altered DNA methylation [35–40], there has been limited research on measures of the built environment and their potential link to epigenetic variation. In a candidate gene study of older adults, a summary measure taking into account both the neighborhood physical environment (esthetic quality) as well as features like crime and social cohesion was associated with epigenetic variation in multiple stress-related genes [41]. The associations observed in this study were independent of both individual measures of socioeconomic status (e.g., employment and education) and perceptions of the neighborhood (including social cohesion), indicating that objective neighborhood characteristics may impact health biomarkers independent of perception and individual socioeconomic status. This observation contrasts with some research suggesting that objective neighborhood quality measures may be largely associated with health outcomes through subjective perception of the neighborhood [8, 9, 42]. However, these previous studies focused mostly only overt (and often self-reported) health outcomes and not molecular measures which may reflect underlying molecular alterations which precede overt health outcomes.

As DNA methylation is an individual measure, it does necessitate that there be some individual-acting mechanism linking neighborhood characteristics and altered DNA methylation. There are multiple individual-acting mechanisms which may account for associations with area-level characteristics including induced stress, exposure to environmental conditions unique to socioeconomically disadvantaged neighborhoods, and limited access to resources such as quality groceries and healthcare. Some of these mechanisms have existing evidence as a means by which neighborhood characteristics can impact individual-level measures [43, 44]. Other factors which may impact these associations are individual-level behavioral factors such as smoking. Though we did control for individual-level behavioral factors like smoking in the models, there may still be components of this mechanism which impact associations through variables not included in the models, e.g., time since quitting. Though the correlation between PC7 and cell counts was low (Supplemental Table 5), adjustment for cell counts did substantially attenuate associations. Interestingly, this attenuation was not observed in neighborhoods with below median levels of large mature trees (Supplemental Table 6). This could indicate that inflammation, reflected in changes in inflammatory cell counts, could be a potential mediating mechanism, but that other mechanisms still persist in neighborhoods derived of greenspace. Given the small sample size, it is beyond the means of this study to fully explore these potential mechanisms, but they should be investigated in future studies to provide a deeper understanding of the mechanisms by which neighborhood characteristics impact health.

Some of the strengths of this study include the robust assessment of objective neighborhood characteristics by trained assessors as opposed to self-report by the residents. This allowed perceptions of the neighborhood by those residing there to be separated from objective neighborhood characteristics. A limitation of the study is that some variables which may modify associations observed were not collected, such as specific occupation. If certain occupational exposures clustered in neighborhoods, then this could confound or modify associations. However, the clustering would have to fall along the lines of the principal components, and the eMRS would have to be associated with the specific occupations which cluster in neighborhoods—which has yet to be shown. Future studies should investigate both the association between the eMRS and occupational exposures as well as attempt to account for occupation should such associations be found. A limitation of the study is the small sample size. Given the limited sample size, we focused on a relevant DNA methylation-based biomarker, as opposed to genome-wide variation, and utilized the continuous form of the eMRS, to maximize power. The limited sample size also impacted our ability to investigate potentially relevant stratifications in this cohort, and could not, for example, study the joint impact of large mature trees and community gardens as < 25 participants had positive indications for both these measures. However, this study is strengthened by using a biomarker derived in an independent sample, which prevents a potential increase in false positives arising from using the same samples to both derive a biomarker and then use that biomarker for analyses. Though the eMRS has not been evaluated in a primarily African-American sample, it has shown strong and consistent prediction in three independent cohorts [27, 31] and similar epigenetic biomarkers have shown validity across ethnicities [17, 28]. Additionally, though this study focused on residents of a single urban area, the epigenetic mortality risk score has been validated in residents of urban areas in Europe and the USA [27, 31].

## Conclusions

In conclusion, we observed that a neighborhood profile highlighted by abandoned cars, poor streets, and non-art graffiti is positively associated with an epigenetic biomarker of mortality risk in an urban population. This study provides molecular evidence of the biological impact of residential neighborhood characteristics and supports research showing that neighborhood characteristics may alter intrinsic biological processes such as the methylation of DNA. As alterations in DNA methylation may be a means by which neighborhood characteristics translate into health disparities, researchers should continue to explore DNA methylation biomarkers and individual loci to more fully understand the biological and socioeconomic implications of these associations.

## Methods

### Cohort

The Detroit Neighborhood Health Study (DNHS) is a prospective cohort study conducted in the metropolitan area of Detroit, Michigan, and was designed to provide a random sample of the city that reflects the demographics of the city. Study coordinators collected a cohort of adult Detroit residents, with surveys and sample collection beginning in the year 2008 and continuing annually until 2012. Study enrollment and annual assessments were conducted via telephone, and the structured interviews were designed to collect detailed information on participant’s neighborhood perception, alcohol and tobacco usage, social support, and health. Additionally, DNHS participants were offered the opportunity to provide a biospecimen sample at their baseline assessment. Of 1547 DNHS participants, 612 consented to providing a blood sample, and these 612 did not differ significantly in sociodemographic profiles from the entire cohort [45]. Informed consent was obtained from all participants, and the study protocol was approved by the University of Michigan Institutional Review Board (HUM00014138) [46, 47].

As part of the baseline survey, a structured assessment of objective neighborhood characteristics was undertaken by trained assessors. In the assessment, 135 census block groups, defined according to the 2000 Census, were visited by a pair of 26 trained assessors. Between June and July of 2008, assessors evaluated 19 neighborhood characteristics (Supplemental Table 2) using standardized protocols, which were adapted from the New York Social Environmental Study neighborhood assessment instrument [48] to be specific for Detroit. Frequencies of observed neighborhood characteristics were calculated as the percent of street segments within each evaluated census block group with that neighborhood characteristic.

### DNA methylation assessment and calculation of epigenetic mortality risk score

Genome-wide DNA methylation was measured in whole blood (leukocytes)-derived DNA with the Illumina 450K DNA methylation array following previously published methods [45, 47, 49]. Briefly, peripheral blood DNA was obtained via venipuncture. Samples were bisulfite converted using the EZ-96 DNA methylation kit (Zymo Research). Bisulfite converted samples were profiled via the Illumina Infinium 450K DNA methylation array per manufacturer protocols. Sample quality control (QC) included the removal of samples with probe detection call rates < 90% and those with an average intensity value < 50% of the experiment-wide sample median or < 2000 arbitrary units. The R package CpGassoc was used for QC procedures [50]. Additionally, probes with detection *P* values > 0.001 were removed, as were samples with missing data for > 10% of probes. Probes with known SNPs and cross-hybridizing probes [51] were also removed. Probe normalization was undertaken using the beta-mixture quantile normalization method [52] as implemented in the R package wateRmelon [53]. ComBat [54] was used to remove batch effects from assigned chip and assigned chip position for each sample. For use of ComBat, beta values were first converted to *M* values. Batch effects were then removed prior to converting *M* values back to beta values. These QC procedures match the standard procedures for the Detroit Neighborhood Health Study [55, 56]. Of the 612 participants providing blood samples, 179 participants were tested on the Illumina 450K DNA methylation array, and of these, 157 passed all QC metrics and had available data on neighborhood characteristics.

We used a validated epigenetic mortality risk score (eMRS) [27] as a molecular indicator of mortality risk as determined by alterations to DNA methylation. The eMRS assessed the percent methylation at 10 CpGs and was developed on the Illumina Infinium 450K methylation array. Both a continuous score, based on the regression coefficients in the discovery sample, and a categorical score, based on the number of “aberrant” CpGs, were developed by Zhang et al. [27]. For this study, we used the continuous score, as 157 samples limit sample sizes across the categorical score for the analyses. All 10 CpGs for the eMRS were available and passed QC in all 157 DNHS participants available for this analysis. The distribution of the eMRS in the study population is given in Supplemental Figure 1.

### Analytic models

Our primary analysis was to evaluate if objective neighborhood characteristics were associated with the eMRS. Given the limited sample size and correlation among the 19 assessed neighborhood characteristics, we performed a principal component (PC) analysis to reduce the number of tests performed as well as to summarize co-varying neighborhood characteristics into components which might be better reflect relationships with the eMRS. We made the a priori decision to examine the first nine PCs (when ranked by percent variance explained) necessary to explain 90% of the variance in the data for analysis.

We used two models of adjustment based on a priori selection of potential confounders. In the first (full) model, we adjusted for age sex, race (White, African-American, and other), DNHS survey wave for the DNA methylation collection (wave 1 vs wave 2), ever smoking, ever alcohol usage, years spent residing in the neighborhood, education (binary indicator for some college or more), and employment (binary indicator for employed vs unemployed). The second model adjusted for all the terms in the first model but also included indicators of personal neighborhood perception. Binary indicators for whether the person liked their neighborhood (“Somewhat” or “A great deal” versus all other responses) and for whether someone agreed with the statement that their neighborhood was a close-knit or unified neighborhood (“Strongly Agree” or “Somewhat Agree” versus all other responses) were used to capture personal perceptions on the likeability and social cohesion/support of an individual’s neighborhood and were asked of each participant at their baseline visit.

Given previous observations that associations with epigenetic biomarkers may vary based on sex [17, 57], we decided to evaluate sex-stratified associations for those PCs significant in the primary analysis. We also stratified analyses based on indicators of greenspace in neighborhoods, as greenspace may be a potentially protective factor for health outcomes [2, 32]. There were two neighborhood characteristics evaluated by the assessors which indicated the presence of greenspace in a neighborhood, one on the presence of large mature trees and the other on the presence of community gardens in the neighborhood (HQ9 and HQ6, respectively, in Supplemental Table 2). For the presence of large mature trees, we stratified individuals based on the median value for HQ6 (84.2%), i.e., the median the percentage of block groups in a neighborhood with large mature trees. As most individuals (88/157) lived in a neighborhood with no observed community gardens, we stratified this variable on the presence of community gardens vs no observed presence. Histograms of the distribution of large mature trees and community gardens in the neighborhoods are given in Supplemental Figures 2 and 3. We also performed a sensitivity analysis adjusting primary associations, as well as those stratified on greenspace, for immune cell counts. Part of the association between the built neighborhood environment and the eMRS may be driven by blood immune cell counts as these would be indicators of inflammatory status, which is one mechanism that may link the built environment to mortality risk. We estimated blood immune cell counts using the established Houseman method [58] to estimate counts of CD4T cells, CD8T cells, natural killer cells, B cells, monocytes, and granulocytes [59].

Associations for the primary analysis were considered significant at the *P* < 0.006 level to adjust for the nine PCs examined. All analyses were done using R version 3.6 [60].

## Supplementary information

**Additional file 1.** Supplemental Table 1. Loadings for each of the top nine principal components. Supplemental Table 2. Definitions of housing quality indicators. Supplemental Table 3. Associations between housing quality indicators and epigenetic mortality risk score. Supplemental Table 4. Associations between PC7 and CpGs which compose the eMRS stratified on neighborhood greenspace. Supplemental Table 5. Pearson correlation (r2) between DNA methylation-derived cell counts and the neighborhood quality principal components used in the analyses. Supplemental Table 6. Associations between PC7 and the epigenetic mortality risk score after additional adjustment for cell counts. Supplemental Figure 1. Distribution of the epigenetic mortality risk score (eMRS) in Detroit Neighborhood Health Study participants. Supplemental Figure 2. Distribution of the percentage of large mature trees observed in the neighborhoods for the study participants. Supplemental Figure 3. Histogram of the distribution of community gardens observed within the neighborhoods for the study participants.

## Data Availability

Data for this study are available by contacting the corresponding author, but requests are subject to approval by the University of North Carolina Institutional Review Board per study protocols to protect the confidentiality of participants.

## Abbreviations

CpG: Cytosine-phosphate-guanine dinucleotide
DNHS: Detroit Neighborhood Health Study
eMRS: Epigenetic mortality risk score
PC: Principal component
