# Supplementary material for: Epigenetic predictors of all-cause mortality are associated with objective measures of neighborhood disadvantage in an urban population
Source: Clin Epigenetics. 2020 Mar 11;12:44. doi: 10.1186/s13148-020-00830-8 (PMC7065313; doi:10.1186/s13148-020-00830-8)
Supplement: Supplementary file 1 — Additional file 1. Supplemental Table 1. Loadings for each of the top nine principal components. Supplemental Table 2. Definitions of housing quality indicators. Supplemental Table 3. Associations between housing quality indicators and epigenetic mortality risk score. Supplemental Table 4. Associations between PC7 and CpGs which compose the eMRS stratified on neighborhood greenspace. Supplemental Table 5. Pearson correlation (r2) between DNA methylation-derived cell counts and the neighborhood quality principal components used in the analyses. Supplemental Table 6. Associations between PC7 and the epigenetic mortality risk score after additional adjustment for cell counts. Supplemental Figure 1. Distribution of the epigenetic mortality risk score (eMRS) in Detroit Neighborhood Health Study participants. Supplemental Figure 2. Distribution of the percentage of large mature trees observed in the neighborhoods for the study participants. Supplemental Figure 3. Histogram of the distribution of community gardens observed within the neighborhoods for the study participants. [file 13148_2020_830_MOESM1_ESM.docx]

**Supplemental Materials:**

|  | PC1 | PC2 | PC3 | PC4 | PC5 | PC6 | PC7 | PC8 | PC9 |
| --- | --- | --- | --- | --- | --- | --- | --- | --- | --- |
| HQ1 | -0.355 | 0.094 | -0.084 | 0.078 | -0.112 | 0.039 | -0.019 | 0.011 | 0.209 |
| HQ2 | -0.362 | 0.097 | -0.005 | -0.046 | -0.133 | 0.016 | 0.104 | 0.157 | 0.033 |
| HQ3 | -0.288 | 0.176 | -0.201 | 0.120 | -0.150 | 0.151 | -0.035 | -0.127 | 0.418 |
| HQ4 | -0.352 | -0.027 | 0.085 | -0.144 | 0.119 | 0.082 | -0.194 | 0.236 | 0.133 |
| HQ5 | -0.037 | -0.163 | -0.375 | 0.160 | 0.368 | 0.571 | 0.027 | -0.024 | -0.249 |
| HQ6 | -0.056 | 0.028 | 0.128 | 0.734 | 0.293 | -0.238 | 0.050 | -0.293 | 0.283 |
| HQ7 | -0.234 | -0.193 | 0.193 | 0.346 | -0.294 | 0.100 | 0.215 | 0.302 | -0.135 |
| HQ8 | 0.332 | 0.056 | -0.001 | 0.207 | -0.171 | -0.094 | 0.191 | -0.088 | -0.248 |
| HQ9 | 0.116 | 0.306 | 0.003 | 0.057 | 0.550 | -0.156 | 0.087 | 0.652 | 0.054 |
| HQ10 | 0.008 | -0.442 | 0.143 | -0.078 | 0.274 | 0.126 | 0.097 | 0.003 | 0.142 |
| HQ11 | -0.291 | 0.086 | 0.114 | 0.191 | 0.226 | -0.142 | -0.202 | -0.147 | -0.520 |
| HQ12 | -0.325 | 0.194 | 0.035 | -0.066 | 0.092 | 0.091 | 0.008 | -0.067 | -0.054 |
| HQ13 | 0.021 | -0.466 | 0.066 | -0.001 | 0.166 | 0.183 | -0.162 | -0.149 | 0.148 |
| HQ14 | -0.053 | -0.429 | -0.015 | 0.073 | -0.099 | -0.194 | 0.437 | 0.282 | 0.132 |
| HQ15 | -0.232 | 0.089 | -0.198 | -0.258 | 0.209 | -0.117 | 0.706 | -0.330 | -0.130 |
| HQ16 | 0.284 | 0.209 | -0.240 | 0.000 | 0.053 | 0.065 | 0.087 | -0.073 | 0.388 |
| HQ17 | -0.062 | -0.197 | -0.495 | -0.082 | 0.118 | -0.310 | -0.166 | 0.023 | 0.055 |
| HQ18 | -0.121 | -0.228 | -0.399 | 0.030 | -0.077 | -0.490 | -0.234 | 0.031 | -0.112 |
| HQ19 | 0.079 | 0.071 | -0.459 | 0.316 | -0.233 | 0.270 | 0.040 | 0.221 | -0.150 |

**Supplemental Table 1.** Loadings for each of the top nine principal components.

See **Supplemental Table 2** for definitions of each housing quality indicator

| Housing Quality Indicator | Metric as Evaluated by Trained Assessor | Mean | SD |
| --- | --- | --- | --- |
| HQ1 | Are there any buildings with broken windows, boarded up windows, or boarded up doors? Percent of sampled block group segments within Neighborhood that have "Yes" for this question. | 34.2 | 14.1 |
| HQ2 | Are there any buildings with outside damage that can only be corrected by major repairs such as siding, shingles, boards, brick, concrete, and stucco? Percent of sampled block group segments within Neighborhood that have "Yes" for this question. | 29.7 | 14 |
| HQ3 | Are there any entirely vacant buildings? Percent of sampled block group segments within Neighborhood that have "Yes" for this question. | 34 | 11.8 |
| HQ4 | Are there any empty, vacant lots? Percent of sampled block group segments within Neighborhood that have "Yes" for this question. | 32.6 | 21.4 |
| HQ5 | Are there any construction sites? Percent of sampled block group segments within Neighborhood that have "Yes" for this question. | 2.4 | 2.5 |
| HQ6 | Is there a community garden? Percent of sampled block group segments within Neighborhood that have "Yes" for this question. | 0.566 | 0.873 |
| HQ7 | Is there graffiti (non-art)? Percent of sampled block group segments within Neighborhood that have "Yes" for this question. | 17.2 | 10.3 |
| HQ8 | Are the street and sidewalk clean? Percent of sampled block group segments within Neighborhood that have "Yes" for this question. | 73.3 | 14.1 |
| HQ9 | Are there any big, mature trees? Percent of sampled block group segments within Neighborhood that have "Yes" for this question. | 83.1 | 9.64 |
| HQ10 | Is there heavy traffic volume? Percent of sampled block group segments within Neighborhood that have "Yes" for this question. | 30.2 | 10.8 |
| HQ11 | Is the street in poor condition? Percent of sampled block group segments within Neighborhood that have "Yes" for this question. | 33 | 11.4 |
| HQ12 | Is the sidewalk in poor condition? Percent of sampled block group segments within Neighborhood that have "Yes" for this question. | 58.7 | 15.2 |
| HQ13 | Is the street noisy? Percent of sampled block group segments within Neighborhood that have "Yes" for this question. | 26.9 | 10.3 |
| HQ14 | Are there people visible on the street? Percent of sampled block group segments within Neighborhood that have "Yes" for this question. | 13.7 | 8.68 |
| HQ15 | Are there any abandoned cars? Percent of sampled block group segments within Neighborhood that have "Yes" for this question. | 6.57 | 4.04 |
| HQ16 | Are any of the following signs visible? A. Neighborhood or Crime Watch. B. Security warning signs. Percent of sampled block group segments within Neighborhood that have "Yes" for this question. | 51.8 | 14.4 |
| HQ17 | Are there any tobacco product advertising signs visible? Percent of sampled block group segments within Neighborhood that have "Yes" for this question. | 1.65 | 1.88 |
| HQ18 | Are there any alcohol advertising signs visible? Percent of sampled block group segments within Neighborhood that have "Yes" for this question. | 2.49 | 2.01 |
| HQ19 | Are there any For Sale OR For Lease OR For Rent signs visible? Percent of sampled block group segments within Neighborhood that have "Yes" for this question. | 18.6 | 6.72 |

**Supplemental Table 2.** Definitions of housing quality indicators

The definitions of each housing quality indicator as assessed by the trained assessors for the Detroit Neighborhood Health Study. A select number of census block groups in each neighborhood of Detroit, Michigan was visited by at least two trained assessors who assessed each of the 19 above neighborhood quality indicators. The final value was computed as the percentage of assessed block groups within each neighborhood with a positive indication for each indicator. Given beside each indicator are the mean and standard deviation (SD) in the Detroit Neighborhood Health Study participants used in this analysis

|  | Effect Estimate | SE | P | LCI | UCI |
| --- | --- | --- | --- | --- | --- |
| HQ1 | -2.8x10^-4^ | 0.003 | 0.92 | -0.01 | 0.01 |
| HQ2 | -0.001 | 0.003 | 0.68 | -0.01 | 0.004 |
| HQ3 | -0.01 | 0.003 | 0.07 | -0.01 | 4.5x10^-4^ |
| HQ4 | -0.002 | 0.002 | 0.20 | -0.01 | 0.001 |
| HQ5 | 3.3x10^-4^ | 0.01 | 0.98 | -0.03 | 0.03 |
| HQ6 | 0.03 | 0.04 | 0.48 | -0.05 | 0.12 |
| HQ7 | 0.004 | 0.004 | 0.25 | -0.003 | 0.01 |
| HQ8 | 0.002 | 0.003 | 0.56 | -0.004 | 0.01 |
| HQ9 | -4.0x10^-4^ | 0.004 | 0.92 | -0.01 | 0.01 |
| HQ10 | 0.001 | 0.004 | 0.83 | -0.01 | 0.01 |
| HQ11 | -0.01 | 0.003 | 0.03 | -0.01 | -0.001 |
| HQ12 | -0.003 | 0.002 | 0.18 | -0.01 | 0.002 |
| HQ13 | 0.003 | 0.004 | 0.39 | -0.004 | 0.01 |
| HQ14 | 0.01 | 0.004 | 0.20 | -0.003 | 0.01 |
| HQ15 | 0.004 | 0.01 | 0.70 | -0.01 | 0.02 |
| HQ16 | 3.5x10^-4^ | 0.003 | 0.89 | -0.005 | 0.01 |
| HQ17 | -0.04 | 0.02 | 0.07 | -0.08 | 0.003 |
| HQ18 | -0.02 | 0.02 | 0.30 | -0.06 | 0.02 |
| HQ19 | -0.01 | 0.01 | 0.18 | -0.02 | 0.003 |

**Supplemental Table 3.** Associations between housing quality indicators and epigenetic mortality risk score. HQ[1-19] = housing quality indicators 1 - 19 (see **Supplemental Table 2** for definitions); LCI = lower 95% confidence interval; SE = standard error; UCI = upper 95% confidence interval

| Stratification | Outcome | Beta | SE | P | LCI | UCI |
| --- | --- | --- | --- | --- | --- | --- |
| None | cg01612140 | -0.03 | 0.01 | 4.5x10^-4^ | -0.04 | -0.01 |
| No Gardens | cg01612140 | -0.03 | 0.01 | 0.002 | -0.05 | -0.01 |
| Tress (Low) | cg01612140 | -0.04 | 0.01 | 2.1x10^-5^ | -0.06 | -0.02 |
| Gardens | cg01612140 | -0.03 | 0.01 | 0.04 | -0.06 | -0.002 |
| Tress (High) | cg01612140 | -0.005 | 0.01 | 0.69 | -0.03 | 0.02 |
| None | cg08362785 | 0.01 | 0.003 | 4.0x10^-4^ | 0.01 | 0.02 |
| No Gardens | cg08362785 | 0.01 | 0.004 | 0.001 | 0.01 | 0.02 |
| Tress (Low) | cg08362785 | 0.02 | 0.004 | 1.0x10^-4^ | 0.01 | 0.03 |
| Gardens | cg08362785 | 0.01 | 0.01 | 0.17 | -0.004 | 0.03 |
| Tress (High) | cg08362785 | 0.01 | 0.01 | 0.37 | -0.01 | 0.02 |
| None | cg10321156 | -0.01 | 0.01 | 0.02 | -0.03 | -0.002 |
| No Gardens | cg10321156 | -0.02 | 0.01 | 0.01 | -0.03 | -0.004 |
| Tress (Low) | cg10321156 | -0.03 | 0.01 | 0.001 | -0.04 | -0.01 |
| Gardens | cg10321156 | -0.01 | 0.01 | 0.65 | -0.04 | 0.02 |
| Tress (High) | cg10321156 | 0.002 | 0.01 | 0.86 | -0.02 | 0.02 |
| None | cg23665802 | -0.02 | 0.01 | 0.001 | -0.03 | -0.01 |
| No Gardens | cg23665802 | -0.02 | 0.01 | 0.01 | -0.04 | -0.01 |
| Tress (Low) | cg23665802 | -0.03 | 0.01 | 1.0x10^-4^ | -0.04 | -0.02 |
| Gardens | cg23665802 | -0.02 | 0.01 | 0.14 | -0.04 | 0.005 |
| Tress (High) | cg23665802 | -0.01 | 0.01 | 0.17 | -0.03 | 0.01 |
| None | cg24704287 | -0.02 | 0.01 | 0.002 | -0.03 | -0.01 |
| No Gardens | cg24704287 | -0.02 | 0.01 | 0.02 | -0.03 | -0.004 |
| Tress (Low) | cg24704287 | -0.03 | 0.01 | 4.6x10^-4^ | -0.04 | -0.01 |
| Gardens | cg24704287 | -0.02 | 0.01 | 0.15 | -0.04 | 0.01 |
| Tress (High) | cg24704287 | -0.01 | 0.01 | 0.38 | -0.03 | 0.01 |
| None | cg25983901 | -0.01 | 0.01 | 0.02 | -0.02 | -0.003 |
| No Gardens | cg25983901 | -0.01 | 0.01 | 0.21 | -0.02 | 0.005 |
| Tress (Low) | cg25983901 | -0.02 | 0.01 | 0.002 | -0.04 | -0.01 |
| Gardens | cg25983901 | -0.01 | 0.01 | 0.32 | -0.03 | 0.01 |
| Tress (High) | cg25983901 | -0.004 | 0.01 | 0.67 | -0.02 | 0.01 |

**Supplemental Table 4**. Associations between PC7 and CpGs which compose the eMRS stratified on neighborhood greenspace.

For those epigenetic loci (CpGs) which composed the epigenetic mortality risk score (eMRS) and had at least a nominal association with PC7 (P < 0.05) we further stratified associations on measures of greenspace to observe if there was a protective effect of neighborhood greenspace. Among individuals residing in neighborhoods with community gardens (Gardens) or above median levels of large, mature trees (Trees (High)) associations between epigenetic loci and PC7 were substantially attenuated and primarily null. The same was true for individuals residing in neighborhoods with a community garden (Gardens). Associations were often strongest for individuals residing in neighborhoods with below median levels of large, mature trees (Trees (Low)). LCI = lower 95% confidence interval, UCI = upper 95% confidence interval

|  | B cells | Monocytes | Granulocytes | CD4T | CD8T | Natural Killer |
| --- | --- | --- | --- | --- | --- | --- |
| PC1 | 0.001333 | 0.000601 | 0.000495 | 1.32E-08 | 0.00022 | 0.000878 |
| PC2 | 0.011954 | 0.001329 | 0.00115 | 0.000838 | 0.002989 | 0.009037 |
| PC3 | 0.001873 | 0.00266 | 0.009881 | 0.045435 | 0.002036 | 0.000129 |
| PC4 | 0.003264 | 0.004372 | 0.000341 | 0.0034 | 0.007228 | 0.001926 |
| PC5 | 0.011936 | 0.000286 | 0.006097 | 0.004535 | 0.009069 | 0.024176 |
| PC6 | 0.001355 | 1.66E-05 | 0.001211 | 0.003617 | 0.005022 | 0.001404 |
| PC7 | 0.035442 | 5.94E-05 | 0.037177 | 0.074293 | 0.001704 | 0.001157 |
| PC8 | 0.000288 | 0.002499 | 0.00565 | 0.001853 | 0.00068 | 0.013005 |
| PC9 | 0.000125 | 0.003121 | 0.001639 | 0.000866 | 0.010259 | 8.60E-05 |

**Supplemental Table 5.** Pearson correlation (r^2^) between DNA methylation-derived cell counts and the neighborhood quality principal components used in the analyses

| Stratification | Beta | P | 95% Confidence Interval |
| --- | --- | --- | --- |
| None | 0.01 | 0.71 | (-0.06, 0.09) |
| Below Median Percentage of Large Mature Trees | 0.10 | 0.04 | (0.01, 0.19) |
| No Community Gardens | -0.002 | 0.97 | (-0.09, 0.09) |

**Supplemental Table 6.** Associations between PC7 and the epigenetic mortality risk score after additional adjustment for cell counts. Models were adjusted for age sex, race, Detroit Neighborhood Health Study survey wave for the DNA methylation collection (Wave 1 vs Wave 2), ever smoking, ever alcohol usage, years spent residing in the neighborhood, education, employment, CD4T cells, CD8T cells, Natural Killer cells, B cells, Monocytes, and Granulocytes. The median level of large mature trees across all study participants was 84.2%.


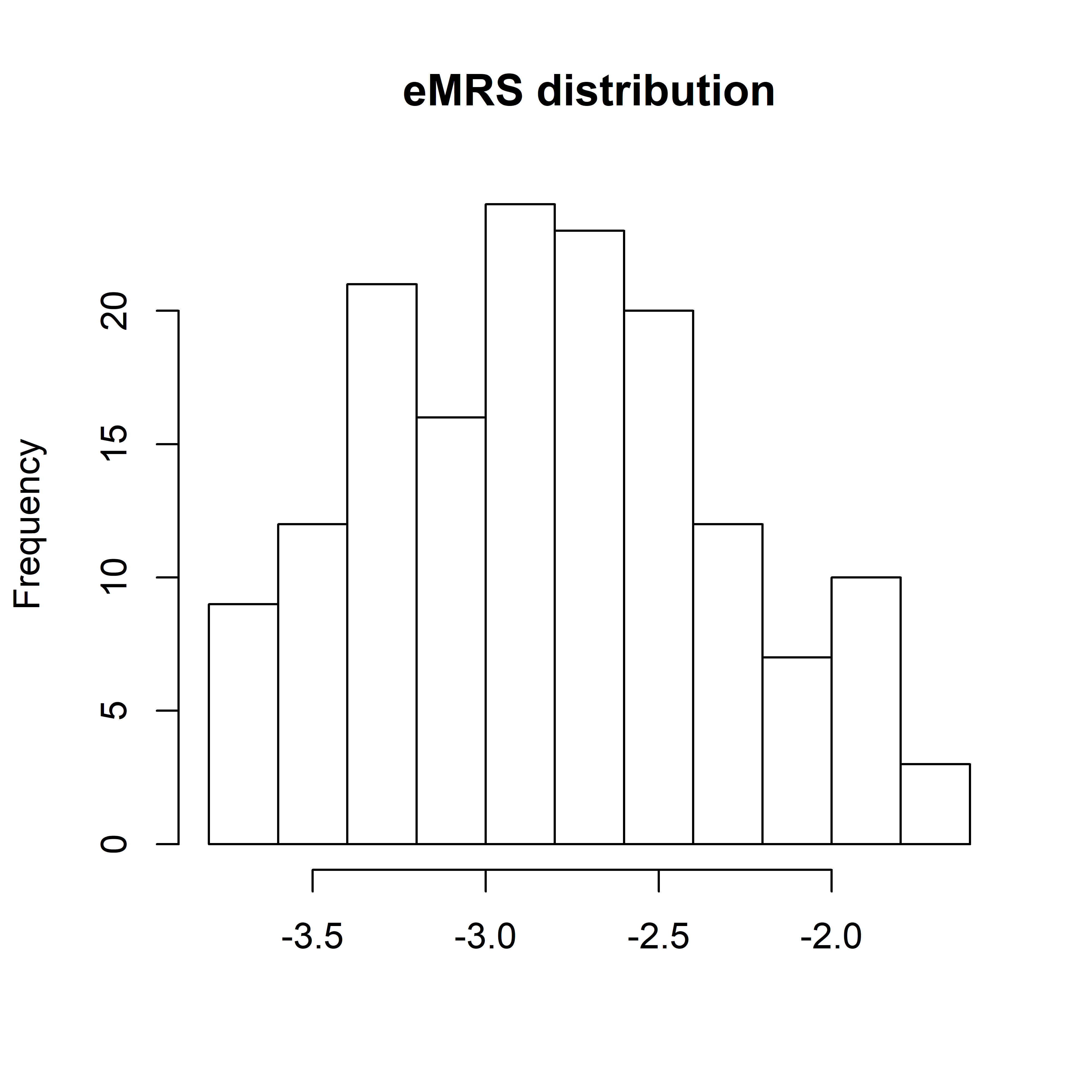


**Supplemental Figure 1.** Distribution of the epigenetic mortality risk score (eMRS) in Detroit Neighborhood Health Study participants.


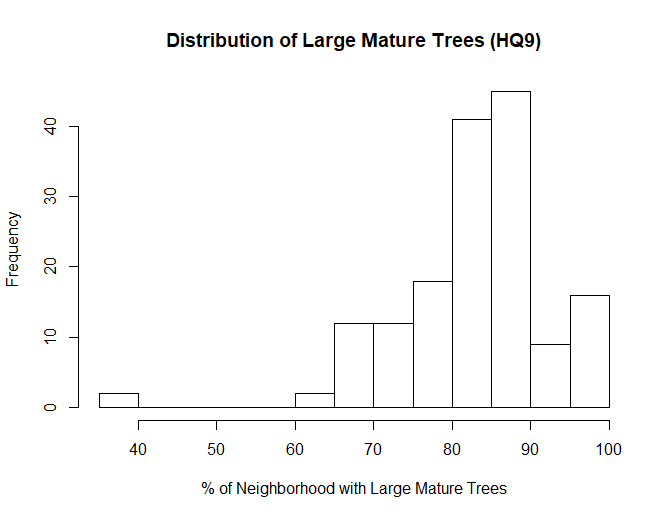


**Supplemental Figure 2.** Distribution of the percentage of large mature trees observed in the neighborhoods for the study participants. The percentage of the neighborhood with large mature trees was calculated as the percentage of street segments within evaluated census block group segments in each neighborhood where large mature trees were observed by trained neighborhood assessors.


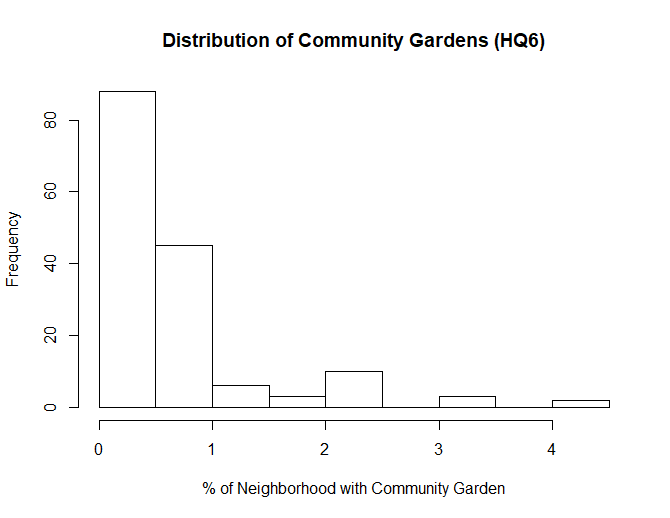


**Supplemental Figure 3**. Histogram of the distribution of community gardens observed within the neighborhoods for the study participants. The percentage of the neighborhood with community gardens was calculated as the percentage of street segments in evaluated census block groups within each neighborhood where community gardens were observed by trained neighborhood assessors.
